# Les conduites addictives en anesthésie réanimation: à propos d un cas

**Published:** 2011-03-22

**Authors:** Issam Serghini, Hicham Anass El Jalil, Sidi Mohamed Hanafi, Abdelkrim Mahmoudi

**Affiliations:** 1 Service d Anesthésie Réanimation, Hôpital Militaire Moulay Ismail, Meknés, Maroc

**Keywords:** Addiction, toxicomanie, anesthésie, Maroc

## Abstract

L′anesthésie et la réanimation sont des métiers difficiles, faits de contraintes et de stress. En milieu anesthésique, des agents toxicomanogènes sont à la disposition des praticiens pour leur usage professionnel. L'exposition quotidienne, la facilité d'acquisition et d'utilisation de ces agents est un danger aux médecins et infirmiers, susceptibles de développer une toxicomanie. L'addiction ou toxicomanie aux agents anesthésiques ne représente qu'une partie du problème des dépendances chimiques qui incluent, les drogues licites que sont le tabac et l'alcool, mais aussi d'autres agents de la pharmacopée comme les antidépresseurs et les sédatifs. Nous rapportons le cas d'un infirmier anesthésiste, ayant des antécédents psychiatriques, toxicomane, admis au service d'accueil des urgences de l’hôpital militaire Moulay Ismail de Meknés dans un tableau de choc hémorragique compliquant un faux anévrysme rompu de l’artère fémorale commune droite.

## Introduction

L’addiction se caractérise par la dépendance, c’est-à-dire, l’impossibilité répétée de contrôler un comportement et la poursuite de ce comportement en dépit de la connaissance des conséquences négatives. Une conduite addictive peut être liée à des produits: alcool, tabac, drogues ou médicaments [4]. La prévention joue un rôle essentiel dans la prise en charge des conduites addictives à des produits. En France, de nombreux efforts sont et ont été déjà faits dans le domaine de la prévention primaire. Ils restent malheureusement insuffisants. La particularité de l’addiction en milieu anesthésique est l’usage des opiacés par voie intraveineuse [1-3]. Ainsi, la prévention secondaire, tel que le dépistage des consommateurs à risque, devient un recours important, tout particulièrement dans le domaine professionnel [5]. À travers ce travail, nous en rapportons un cas.

## Patient et observation

**Figure 1:**
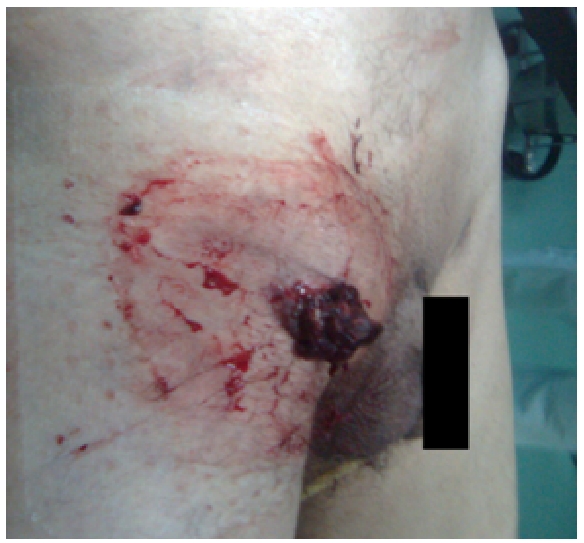
Le faux anévrysme septique de l’artère fémorale commune droite rompu

Il s’agit d’un homme de 58 ans, infirmier anesthésiste de fonction, tabagique chronique, suivi depuis 20 ans en psychiatrie pour stress post traumatique ayant comme habitus toxiques des conduites toxicophiles depuis 15 ans (appétences aux benzodiazépines par voie intraveineuse au niveau fémorale), admis au service accueil des urgences de l’hôpital militaire Moulay Ismail de Meknés pour un faux anévrysme septique de l’artère fémorale commune droite rompu (Figure 1).

L’évaluation clinique à l’admission retrouvait chez ce patient un saignement actif de grande abondance au niveau de la région de Scarpa motivant son admission en urgence en salle de déchoquage, associé à plusieurs détresses vitales: trouble de la conscience avec un score de Glasgow à 9, un état de choc avec une fréquence cardiaque à 120 battements/min, une PA à 90/50 mmHg. L’état respiratoire était également précaire: polypnée, SpO2 à 91 % à l’air ambiant ; le tout évoluant dans un contexte fébrile à 38,5°. L’examen du membre inférieur droit trouve un membre froid cyanosé, une paralysie sensitivo-motrice avec abolition des pouls distaux. Nous avons aussi noté que toutes les veines des deux membres supérieurs étaient éclatées.

En résumé, ce malade présentait un tableau de choc hémorragique compliqué de détresse neurologique sur rupture d’un faux anévrysme septique de l’artère fémorale commune droite. La prise en charge initiale consistait en une pose de deux voies veineuses périphériques 14 et 16 Gauge, un remplissage vasculaire par 1 litre de sérum salé isotonique, une oxygénothérapie à 6 litres/minute au masque à haute concentration. Une voie centrale a été placée en jugulaire interne. Cette stratégie a permis de restaurer une pression artérielle moyenne à 80 mmHg. La biologie initiale objective une anémie à 6,4 g d’Hb/dl, un hématocrite à 21,6 %, plaquettes 265 000 plaquettes/mm3, un taux de prothrombine à 66 %, une acidose tissulaire manifeste avec des lactates à 11 mmol/l. Après la mise en condition le chirurgien vasculaire a été contacté.

Au bloc opératoire sous anesthésie générale après une crush induction par 2mg d’ hypnovel, 20 mg d’ étomidate et 40 mg d’ esméron avec application de la manœuvre de Sellick, le patient est intubé par un tube endotrachéal de 7,5mm et mis sous ventilation artificielle. L’analgésie est assurée par 250 mcg de fentanyl et l’entretien de l’anesthésie est réalisée par de l’halothane 100 % avec un mélange équimolaire oxygène /protoxyde d’azote.

**Figure 2:**
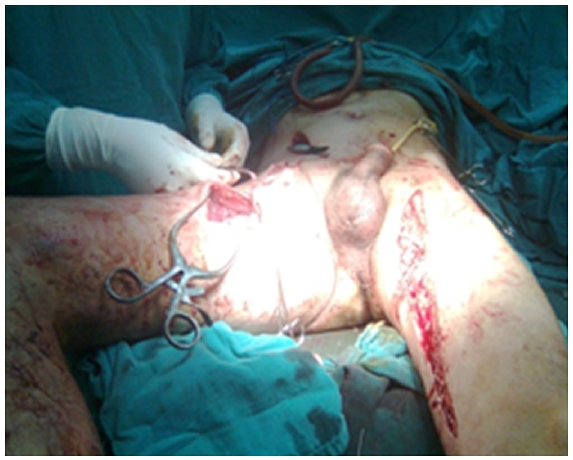
L’abord de l’artère iliaque externe droite par voie rétro péritonéale pour clampage de celle-ci avec mise à plat de l’anévrysme fémoral au niveau du Scarpa droit et ligature du bout proximal et distal de l’artère fémorale commune puis parage de la zone nécrosée

En per opératoire patient a été transfusés par 04 culots globulaires iso-groupe iso-rhésus à l’aide d’un accélérateur et réchauffeur de transfusion. En premier temps un abord de l’artère iliaque externe droite par voie rétro péritonéale a permis le clampage de celle ci pour contrôler l’hémorragie avec une mise à plat de l’anévrysme fémoral au niveau du Scarpa droit et ligature du bout proximal et distal de l’artère fémorale commune a été réalisé avec parage de la zone nécrosé (Figure 2). Le deuxième temps consistait à la réalisation d’un pontage croisé en transpérinéal en veine saphène interne gauche. L’artériographie de contrôle en fin d’intervention a montré une bonne perméabilité du pontage. Les résultats de l’analyse cytobactériologique pus et collections purulentes après ponction sur seringue: Examen direct BGN culture: culture positive après 24 h met en évidence un Proteus Mirabilis sensible à la céfotaxime.

En postopératoire le malade est mis sous céfotaxime (1g/8 h) et calciparine (0,3 ml/8h) paracétamol (1g/6h). A J4, l’examen du membre inférieur droit du patient trouve une ischémie consommée nécessitant une amputation du tiers moyen de la jambe droite sous rachianesthésie.

## Discussion

Une forte prédominance masculine est notée chez les anesthésistes toxicomanes. Les usagers de substances psycho-actives consomment régulièrement plusieurs types de substances, dont l’une d’entre elles est prédominante. Les substances consommées sont, outre les opiacés, les benzodiazépines, les neuroleptiques, le protoxyde d’azote, la kétamine et même le propofol. Toutes ces substances font courir un risque vital aux toxicomanes en raison du risque de surdosage ou d’erreur d’administration (par exemple: injection de 10 fois la dose habituelle ou injection de curares par inadvertance). Cet usage répété conduit à une dépendance et à une tolérance, de telle sorte que les quantités détournées par les toxicomanes augmentent avec le temps.

Les études portant sur les profils de personnalité retrouvent des caractéristiques non spécifiques au milieu professionnel (antécédents familiaux de toxicomanie ou de maladie psychiatrique, divorce, difficultés d’insertion professionnelle, etc.) mais qui sont habituellement considérées comme des facteurs de risque [6,7]. Le risque de décès est estimé de 10 à 15 % sur 5 à 10 ans, ce qui est considérable. Les décès peuvent être liés à une overdose accidentelle ou volontaire [8,9].

La toxicomanie provoque des troubles du comportement qui ne vont qu’en s’aggravant au fil du temps et conduisent un jour ou l’autre au diagnostic. Le déni de ces comportements et de la toxicomanie est constant chez les toxicomanes et fait partie de la symptomatologie. La liste des anomalies du comportement observées chez les toxicomanes est assez stéréotypée: des changements d’humeur (dépression, anxiété, euphorie) au cours d’une même journée; des absences répétées et inexpliquées; des sorties fréquentes de salle d’opération en cours d’anesthésie; une préférence marquée pour la pratique de l’anesthésie en solitaire; la présence nocturne à l’hôpital en dehors des périodes de garde; des absences réitérées de réponse aux appels pendant les gardes; l’allégation de problèmes de santé multiples, personnels ou familiaux justifiant éventuellement « l’emprunt » d’opiacés. Les infirmiers ayant des conduites addictives avec les agents anesthésiques détournent à leur profit les agents normalement administrés aux patients. En témoignent, des négligences répétées quant au relevé de l’information reportée sur les feuilles d’anesthésie, le fait que les patients présentent des douleurs postopératoires excessives ou inhabituelles ou des signes de réveil au cours de l’anesthésie, ou le fait que ces patients aient une prescription d’analgésique sans commune mesure avec la douleur attendue. Les comportements décrits ne sont pathologiques que du fait de leur répétition. Les causes de la toxicomanie sont multiples, les facteurs liés au parcours personnel de chaque individu sont bien évidemment au-delà des mesures de prévention collective. Il est à l’inverse possible que les conditions de travail et l’environnement favorisent l’évolution vers la toxicomanie [6].

D’une façon plus générale, si l’on admet que des facteurs liés aux conditions de travail peuvent favoriser la toxicomanie, toutes les initiatives visant à baisser le niveau des pressions et des contraintes sont bienvenues, notamment celles qui visent à réduire les conditions d’exercice dans l’isolement. Malgré la réitération de ces comportements, le délai écoulé entre le début de la toxicomanie et son identification est souvent long, de plusieurs mois à plusieurs années. La découverte est souvent fortuite soit à l’occasion de procédures routinières de contrôle des stocks médicamenteux.

## Conclusion

Le toxicomane en milieu professionnel est une personne en danger. La mise en évidence d’une addiction en milieu anesthésique est difficile et se fait sur des anomalies du comportement professionnel. Les agents utilisés sont multiples et souvent associés entre eux. Les conduites addictives aux agents anesthésiques sont plus fréquentes chez le personnel en formation. Les médecins et infirmiers toxicomanes nécessitent une prise en charge en milieu spécialisé qui devrait leur permettre à terme une réinsertion et une réorientation professionnelles.

## Conflits d’ intérêt

Les auteurs ne déclarent aucun conflit d´intérêts

## Contribution des auteurs

Tous les auteurs ont également contribué à ce travail et ont lu et approuvé la version finale du manuscrit.
